# Is there any association between type of dietary fat and quality of life in hemodialysis patients? A cross-sectional study

**DOI:** 10.3389/fnut.2024.1430595

**Published:** 2024-09-26

**Authors:** Fatemeh Navab, Sahar Foshati, Mahdi Vajdi, Gholamreza Askari, Firouzeh Moeinzadeh, Houri Heshamtipour, Soheila Mirzaeian, Mohammad Hossein Rouhani

**Affiliations:** ^1^Student Research Committee, Nutrition and Food Security Research Center and Department of Community Nutrition, School of Nutrition and Food Science, Isfahan University of Medical Sciences, Isfahan, Iran; ^2^Nutrition Research Center, Department of Clinical Nutrition, School of Nutrition and Food Sciences, Shiraz University of Medical Sciences, Shiraz, Iran; ^3^Nutrition and Food Security Research Center and Department of Community Nutrition, School of Nutrition and Food Science, Isfahan University of Medical Sciences, Isfahan, Iran; ^4^Isfahan Kidney Diseases Research Center, Isfahan University of Medical Sciences, Isfahan, Iran; ^5^St Boniface Hospital, University of Manitoba, Winnipeg, MB, Canada

**Keywords:** dietary fat, fatty acids, quality of life, hemodialysis, cross-sectional study

## Abstract

**Background:**

Hemodialysis (HD) patients have a low quality of life (QOL), and dietary intakes may impact both somatic and psychosocial aspects of QOL. Nevertheless, the relationship between QOL and different dietary fats has not yet been evaluated.

**Objective:**

The purpose of this study was to assess the association between QOL and the types/quantities of dietary fats intake in HD patients.

**Methods:**

In this multi-center cross-sectional study, 251 adult patients under dialysis for at least 3 months were included. Participants’ dietary intakes were collected using a validated 168-item semi-quantitative FFQ during the past year. Moreover, to assess QOL, Kidney Disease Quality of Life Short Form (KDQOL-SF 1/3) was used. The linear regression between QOL and different types of dietary fats was conducted. *p* < 0.05 was statistically significant.

**Results:**

Overall, 66 women and 185 men participated in our study. Regression analysis adjusted for total calorie intake showed that there was a negative association between QOL and total fat (95% CI: −0.187, −0.043), SFA (95% CI: −0.688, −0.143), MUFA (95% CI: −0.389, −0.065) and PUFA (95% CI: −0.401, −0.056) when types of dietary fats were individually included to the regression analysis. When all types of dietary fats were simultaneously entered into the analysis, the association between QOL and MUFA (95% CI: −0.243, 1.031) and PUFA (95% CI: −1.159, 0.084) were attenuated. The regression coefficient for SFA remained significant (95% CI: −0.968, −0.138). Also, there was a marginally significant association between SFA and the risk of low QOL was observed when all types of dietary fats were simultaneously entered into the analysis (OR = 1.051, 95% CI: 0.998–1.104).

**Conclusion:**

Our investigation found a negative association between SFA consumption and QOL among different types of dietary fats. Furthermore, SFA mediated the relationship between QOL, MUFA, PUFA, and total fat. So, modification of dietary fat intake could enhance QOL in HD patients.

## Introduction

Most patients with end-stage renal disease (ESRD) should be under renal replacement therapy such as hemodialysis (HD) (1). Although HD is necessary for survival in these patients, it cannot mimic all kidney functions (2). Therefore, patients suffer from several metabolic dysfunctions that affect their quality of life (QOL). Evidence showed that the physical and psychosocial aspects of QOL in HD patients were not as good as healthy subjects (3, 4). Assessment of QOL can provide a comprehensive medical judgement and promote patient-physician relationships (5). Limited physical activity, emotional distress, increased financial burden, HD’s time-consuming nature, and negative social pressure are the main reasons for poor QOL in HD patients (6). Nevertheless, the importance of malnutrition to QOL should not be neglected (7–9).

HD patients are susceptible to protein-energy malnutrition (PEM) because of poor appetite, decreased dietary intake and loss of nutrients through dialysis membranes (10, 11). More significant morbidity, functional impairment, more prolonged hospitalizations, and decreased QOL are strongly related to PEM (12, 13). Poor quality of life may contribute to eating disorders and consequently lead to malnutrition (14). Alternatively, previous findings indicated that malnutrition negatively affects the QOL of the patients by reducing their muscle strength (15) and affecting psychological and neurological complications (16). As malnourished patients have a worse quality of life, early diagnosis and treatment of malnutrition are crucial (12, 13). Research have shown that PEM is often caused by insufficient dietary energy intake in HD patients (17, 18). Therefore, the intake of sources of dietary energy should be sufficient. Dietary fat is the most energy-dense macronutrient (19). Moreover, fat is considered the main store of energy in the body (20). Therefore, adequate dietary fat intake may prevent PEM and positively affect QOL.

Besides the importance of dietary fat as a source of energy, the profile of dietary fats has a significant role in physiological functions (21). Furthermore, since CKD patients are at high risk of cardiovascular disease, types and amounts of dietary fats should be noticed (22). Meanwhile, nutritional recommendations regarding fat intake in CKD patients have been given less attention. For instance, Kidney Disease Improving Global Outcomes guidelines (KDIGO) did not recommend dietary fat intake in HD patients (23). Moreover, as a specific kidney disease dietary recommendation, the 2020 KDOQI update, only recommended supplementation with long-chain *ω*-3 PUFAs for managing dyslipidemia in kidney disease (24). However, they made no recommendations regarding the dietary intake of these fatty acids. According to KDOQI, 1.3–4 g/d supplementation of long-chain n-3 PUFA is recommended for adults on MHD to lower triglycerides and LDL cholesterol and raise HDL levels (24). In general, Mono-unsaturated fatty acid (MUFA) and polyunsaturated fatty acid (PUFA) are considered healthy dietary fats (25). Conversely, saturated fatty acid (SFA) intake is a major cardiovascular risk factor (26). PUFAs, particularly docosahexaenoic acid (DHA), are concentrated in the neuronal cell membrane (27). These fatty acids are crucial for the functions and development of the nervous system (28). Furthermore, fatty acids contribute to membrane fluidity and function, synaptic transmission and metabolism of neurotransmitters (29–31). A study reported that SFA intake was directly, and consumption of MUFAs and alpha-linolenic acid (ALA) inversely related to anxiety risk (32). Moreover, the beneficial effects of supplementing with n-3 PUFA on improving depressive symptoms and QOL among HD patients were reported (33, 34). In contrast, a meta-analysis showed that n-3 PUFA supplementation had no significant impact on anxiety (35). Therefore, results regarding the association between dietary fat and QOL are inconsistency. Also, there were no studies regarding the relationship between QOL and dietary fats in HD patients. The present study aimed to investigate the association between QOL and the profile of dietary fat intake (i.e., total fat, SFA, MUFA, PUFA, and cholesterol) in HD patients.

## Materials and methods

### Study population and protocol

This multi-center cross-sectional study was conducted on 251 maintenance HD patients from September 2021 to March 2022.Participants were recruited from the five governmental and charity hemodialysis centers in Isfahan, Iran. We included patients if they met the following criteria: undergoing maintenance HD for at least the previous 90 days, being at least 18 years old, and having the ability and willingness to participate in our study. Individuals were excluded if their daily energy intake was above 4,200 kcal/d (17,573 kJ) or less than 800 kcal/d (3,347 kJ) (36). A brief description of the study’s significance, methods, goals, and timeline was provided to all participants. All volunteers filled out the informed consent forms before starting the study. This study was approved by The Research Ethics Committee of Isfahan University of Medical Sciences, Isfahan, Iran (IR.MUI.RESEARCH.REC.1399.605).

### Dietary assessment

A semi-quantitative food frequency questionnaire (FFQ) containing 168 food items was used to assess dietary intakes during the past year. The reliability and validity of this questionnaire were previously evaluated and found to be acceptable in the Iranian population (37). FFQ was completed via face-to-face interviews with a trained dietitian only once at the baseline. For each food item, two parameters were asked: 1) the frequency of consumption (never or < 1 times/month, 1–3 times/month, 1 times/week, 2–4 times/week, 5–6 times/week, 1 times/day, 2–3 times/day, 4–5 times/day and ≥6 times/day) in the previous year, and 2) The usual amounts of food consumed every time were based on a standard portion size. Then mean daily intake was calculated for each food item using the following formula: (frequency of consumption × amount of food item intake (g) / duration of reported frequency (day). For example, in the case of consuming one potato (90 grams) two times per week, the mean daily intake of potato was 2 × 90/7 = 25.7 g/day. Then macro/micronutrients intake were assessed based on the mean daily intake by Nutritionist IV software (First Databank, Hearst Corp).

### Assessment of quality of life

We used the Kidney Disease Quality of Life Short Form (KDQOL-SF 1/3) to assess QOL in HD patients. Combination of SF-36 generic instrument with the kidney disease-specific instrument which forms the KDQOL-SF TM version 1.3. This questionnaire is composed of 80 items arranged into 19 categories. There are 43 items focused on kidney disease in KDQOL-SFTM 1.3. This questionnaire contained 11 domains including a list of symptoms and problems (12 items), the impact of renal disease on daily life (8 items), the burden of renal disease (4 items), occupational status (2 items), cognitive function (3 items), social contacts quality (3 items), sexual function (2 items), sleep quality (4 items), social support (2 items), dialysis staff encouragement (2items), and patient satisfaction (1 items).

SF-36 consists of 8 domains (36 items) that measure functioning and well-being. These domains covered physical functions (10 items), physical roles (4 items), pain (2 items), general health (5 items), emotional health (5 items), psychological roles (3 items), social activities (2 items), and energy/fatigue (4 items). Finally, a 0–10 scale is used for rating respondents’ overall health. A physical component summary (PCS) and a mental component summary (MCS) are further summarized by the SF-36 instrument. Subjects scored from 0 to 100, with higher values indicating better QOL (38). The reliability and validity of this questionnaire have been previously assessed and found to be acceptable in Iranian HD patients (39).

### Anthropometric measurements

Height was measured with 0.1 cm precision using non-stretchable tape. It was measured in a standing position while shoulders and barefoot touching the wall. Dry weight was defined as the minimum tolerable weight achieved after a dialysis session by means of gradual change in post-dialysis weight at which there are no signs or symptoms of either hypovolemia or hypervolemia (40). Dry weight was measured to the nearest 0.1 kg using a calibrated digital floor scale after dialysis session when no signs or symptoms of either hypovolemia or hypervolemia were observed. Also, individuals were asked to wear light clothing without shoes (41). The body mass index (BMI) was determined by dividing dry weight to squared height. Waist circumference (WC) was also measured to the nearest 0.1 cm using a stable tension tape (42). It was measured at the midpoint between the lowest rib and iliac crest in a standing position. Hip circumference (HC) was measured at the maximum circumference over the buttocks to the nearest 0.1 cm (43). By dividing the WC by the HC, the waist-to-hip ratio (WHR) was determined. A flexible, non-stretchable tape was used to measure the mid-upper arm circumference (MUAC). It was measured on the bare left arm between the inferior border of the acromion process (shoulder bone) and the tip of the olecranon process (elbow) to the nearest 0.1 cm (44).

### Assessment of quality of HD

To assess the quality of HD, we used the Urea reduction ratio (URR) and Kt/V. URR was calculated by following (45):


$$\begin{array}{c} URR=\left[\left({\mathrm{Blood Urea}}_{pre-\mathrm{dialysis}}-{\mathrm{Blood Urea}}_{\mathrm{post}-\mathrm{dialysis}}\right)/{\mathrm{Blood Urea}}_{pre-\mathrm{dialysis}}\right]\\ \times 100 \end{array}$$

the dialyzer urea clearance (K) is multiplied by dialysis time to calculate the Kt/V (t) divided by the subject’s urea distribution volume (V) (45).

### Assessment of other variables

Age, place of habitation, marital and occupational status, education, family income, smoking history, menopausal status and alcohol intake were collected by oral questions. Medical records were used to gather data, including medications, dialysis vintage, dialysis frequency, dialysis duration and cause of renal failure.

### Statistical analysis

The normal distribution of dependent variables was assessed by Kolmogorov–Smirnov test and Q-Q plot. Qualitative variables were expressed as numbers (percentages). Quantitative variables were reported as mean ± standard deviation (SD). Comparisons between groups were performed using one-way analysis of variance (ANOVA) and also the Chi-square test, as appropriate. Linear regression was used to investigate the relationship between dietary fat intake and QOL. Logistic regression anlaysis was applied to assess risk of low QOL (median cut) per one-unit increase in different dietary fat intake. The regression coefficient and 95% confidence intervals were reported. Statistical significance was defined as *p* < 0.05. All statistical analyses were performed using SPSS version 21.

## Results

Overall, 66 women and 185 men were included in this study. Mean intake of total fat, cholesterol, SFA, MUFA and PUFA was 58.5 g/d, 201.57 mg/d, 18.86 g/d, 20.18 g/d and 11.85 g/d, respectively. The general characteristics of participants across tertiles of specific types of fat intake are summarized in Table 1. The percentage of men was higher in the last tertile of all types of dietary fats compared with the lower tertiles (*p* < 0.001 for all types of dietary fats). Similarly, subjects in the top tertile of all types of dietary fats had higher weight (*p* = 0.002 for total fat, *p* = 0.02 for cholesterol, *p* = 0.019 for SFA, *p* = 0.001 for MUFA and *p* = 0.023 for PUFA) and height (p < 0.001for total fat, cholesterol, SFA and MUFA, and *p* = 0.008 for PUFA). Individuals in higher tertiles of PUFA were younger than those in the lowest tertile (*p* = 0.038). URR decreased across tertiles of total fat (*p* = 0.005), MUFA (*p* = 0.022), and PUFA (*p* = 0.026). Additionally, subjects in the highest tertile of SFA had less dialysis duration per session than the lowest tertile (*p* = 0.016). Moreover, there were mostly retired patients in the top tertile of all types of dietary fats, whereas most unemployed individuals were in the lowest tertile (*p* < 0.001 for total fat, cholesterol, SFA, MUFA and *p* = 0.01 for PUFA). No significant differences were observed regarding other characteristics throughout the tertiles of different types of dietary fat. Energy-adjusted nutrient intake across tertiles of specific types of dietary fatsis shown in Table 2. Participants with the highest adherence to total fat intake had a higher consumption of energy (*p* < 0.001), protein (*p* < 0.001), vitamin A (*p* < 0.001), vitamin E (*p* < 0.001), calcium (*p* < 0.001), thiamin (*p* = 0.042), riboflavin (*p* < 0.001), niacin (*p* < 0.001), and phosphorus (*p* < 0.001). Moreover, individuals with the highest consumption of cholesterol had a higher intake of energy (*p* < 0.001), protein (*p* < 0.001), vitamin A (*p* = 0.016), vitamin E (*p* < 0.001), calcium (*p* < 0.001), riboflavin (*p* < 0.001), niacin (*p* < 0.001), as well as phosphorus (*p* < 0.001).Patients with higher consumption of SFA consume more energy (*p* < 0.001), protein (*p* < 0.001), vitamin A (*p* < 0.001), vitamin E (*p* < 0.001), calcium (*p* < 0.001), riboflavin (*p* < 0.001), niacin (*p* < 0.001), and phosphorus (*p* < 0.001). Subjects in the top tertile of MUFA had the highest consumption of energy (*p* < 0.001), protein (*p* < 0.001), vitamin A (*p* < 0.001), vitamin E (*p* < 0.001), calcium (*p* < 0.001), riboflavin (*p* < 0.001), niacin (*p* = 0.002), and phosphorus (*p* < 0.001). Individuals in the last tertile of PUFA had the highest amounts of energy (*p* < 0.001), protein(*p* = 0.003), niacin (*p* = 0.047), and phosphorus (*p* = 0.042). Other nutrients were not significantly different across tertiles of different dietary fats.

**Table 1:** General characteristics of hemodialysis patients across tertiles of different types of dietary fats.

	Total fat				Cholesterol				Saturated fatty acid				MUFA				PUFA			
Variables	T1 (<40.18 gr/day)	T2 (40.18–58.84 gr/day)	T3 (≥58.84 gr/day)	*p* value	T1 (<132.69 mg/day)	T2 (132.69–213.22 mg/day)	T3 (≥213.22 mg/day)	*p* value	T1 (<12.59 gr/day)	T2 (12.59–20.11 gr/day)	T3 (≥20.11 gr/day)	*p* value	T1 (<13.97 gr/day)	T2 (13.97–20.61gr/day)	T3 (≥20.61gr/day)	*p* value	T1 (<7.49 gr/day)	T2 7.49–11.78 gr/day)	T3 ≥11.78 gr/day)	*p* value
*N*	83	84	84		83	84	84		83	84	84		83	84	84		83	84	84	
Demographic variables																				
Sex (% male)	45.8	83.3	91.7	<0.001	53	73.8	94	<0.001	44.6	88.1	88.1	<0.001	49.4	81	90.5	<0.001	56.6	76.2	88.1	<0.001
Age)y)	61.85 ± 13.98	59.25 ± 14.40	56.48 ± 15.51	0.063	61.30 ± 13.66	57.33 ± 15.02	58.95 ± 15.42	0.218	60.56 ± 13.92	58.00 ± 14.82	59.01 ± 14.75	0.529	62.08 ± 14.07	58.55 ± 14.25	56.95 ± 15.59	0.071	62.09 ± 13.41	59.22 ± 15.56	56.27 ± 14.79	0.038
Marital status (% married)	83.1	88	90.9	0.357	88	82.7	90.9	0.331	88	85.7	87.3	0.916	85.5	87.5	88.4	0.864	84.1	87.5	90.0	0.558
Dry Weight (kg)	62.83 ± 12.78	69.38 ± 15.05	69.52 ± 13.58	0.002	64.06 ± 13.06	67.53 ± 14.48	70.15 ± 14.31	0.02	63.84 ± 13.35	69.80 ± 14.67	68.10 ± 13.84	0.019	62.73 ± 13.07	70.09 ± 14.91	68.90 ± 13.38	0.001	63.78 ± 12.68	68.93 ± 14.34	69.03 ± 14.80	0.023
Height (cm)	161.21 ± 8.94	164.91 ± 8.45	168.80 ± 8.54	<0.001	160.75 ± 7.87	165.69 ± 9.62	168.48 ± 8.23	<0.001	161.36 ± 8.82	166.70 ± 8.71	166.88 ± 8.93	<0.001	161.42 ± 9.05	165.51 ± 8.82	168.01 ± 8.44	<0.001	162.66 ± 8.61	165.29 ± 9.06	167.00 ± 9.35	0.008
Body mass index(kg/m^2^)	24.15 ± 4.44	25.45 ± 4.99	24.30 ± 3.87	0.122	24.82 ± 4.87	24.53 ± 4.80	24.56 ± 3.70	0.900	24.49 ± 4.57	24.98 ± 4.31	24.42 ± 4.58	0.684	24.05 ± 4.56	25.48 ± 4.66	24.37 ± 4.13	0.095	24.10 ± 4.50	25.09 ± 4.12	24.72 ± 4.79	0.353
Waist-Circumference (cm)	95.05 ± 12.50	96.70 ± 13.53	93.86 ± 12.23	0.353	96.25 ± 12.51	94.98 ± 13.14	94.39 ± 12.72	0.632	95.72 ± 12.91	95.22 ± 12.43	94.67 ± 13.09	0.869	94.42 ± 13.11	97.45 ± 13.06	93.73 ± 11.95	0.134	95.28 ± 12.18	95.68 ± 12.91	94.64 ± 13.31	0.870
Hip-Circumference (cm)	98.48 ± 8.98	97.57 ± 9.90	96.54 ± 10.18	0.437	98.87 ± 9.32	96.91 ± 10.16	96.80 ± 9.55	0.301	98.60 ± 8.99	97.54 ± 8.87	96.45 ± 11.06	0.360	97.98 ± 9.55	98.60 ± 9.04	96.00 ± 10.37	0.191	97.83 ± 9.54	97.84 ± 9.55	96.91 ± 10.07	0.778
Arm-Circumference (cm)	28.11 ± 3.58	28.89 ± 4.18	28.10 ± 3.62	0.304	28.58 ± 4.01	28.10 ± 3.90	28.44 ± 3.52	0.703	28.48 ± 3.70	28.55 ± 3.80	28.08 ± 3.95	0.689	27.98 ± 3.68	29.14 ± 4.03	27.98 ± 3.62	0.073	28.00 ± 3.46	29.08 ± 3.90	28.03 ± 3.98	0.112
Dialysis vintage (W)	45.35 ± 35.94	50.10 ± 46.60	45.09 ± 53.35	0.732	42.32 ± 35.24	51.37 ± 46.93	46.92 ± 53.21	0.450	44.25 ± 34.88	47.20 ± 46.42	49.17 ± 54.16	0.787	44.85 ± 36.62	52.43 ± 47.22	43.22 ± 51.99	0.387	45.50 ± 36.70	51.43 ± 44.00	43.60 ± 54.71	0.519
Dialysis frequency (%)																				
1x per week	2.4	0.0	1.2	0.605	2.4	1.2	0.0	0.053	2.4	0.0	1.2	0.835	2.4	0.0	1.2	0.515	2.4	0.0	1.2	0.690
2x per week	16.9	17.8	25		21.7	9.5	28.6		18.1	20.2	21.4		19.3	15.5	25		19.3	16.6	23.8	
3x per week	79.5	79.8	72.6		74.7	88.1	69.0		78.3	77.4	76.2		77.1	83.3	71.4		77.1	81	73.8	
4x per week	1.2	2.4	1.2		1.2	1.2	2.4		1.2	2.4	1.2		1.2	1.2	2.4		1.2	2.4	1.2	
Dialysis duration/session (h)	3.93 ± 0.21	3.95 ± 0.19	3.89 ± 0.30	0.247	3.93 ± 0.24	3.94 ± 0.21	3.90 ± 0.26	0.523	3.92 ± 0.23	3.98 ± 0.12	3.87 ± 0.31	0.016	3.95 ± 0.18	3.94 ± 0.23	3.88 ± 0.29	0.158	3.95 ± 0.17	3.94 ± 0.21	3.88 ± 0.30	0.86
Kt/V	1.35 ± 0.25	1.32 ± 0.21	1.28 ± 0.23	0.142	1.35 ± 0.21	1.30 ± 0.27	1.30 ± 0.21	0.360	1.33 ± 0.26	1.34 ± 0.20	1.28 ± 0.23	0.184	1.35 ± 0.24	1.33 ± 0.21	1.27 ± 0.23	0.053	1.35 ± 0.24	1.30 ± 0.22	1.29 ± 0.23	0.187
URR	0.74 ± 0.18	0.71 ± 0.16	0.67 ± 0.07	0.005	0.72 ± 0.17	0.71 ± 0.17	0.69 ± 0.10	0.484	0.73 ± 0.17	0.71 ± 0.15	0.68 ± 0.11	0.134	0.73 ± 0.16	0.73 ± 0.18	0.67 ± 0.07	0.022	0.71 ± 0.12	0.73 ± 0.21	0.67 ± 0.07	0.026
Cause of renal failure (%)																				
Diabetes mellitus	24.1	25	23.8	0.510	21.7	25	26.1	0.362	24.1	22.6	26.2	0.307	26.5	20.2	26.2	0.735	25.3	22.6	25	0.913
Hypertension	26.5	33.3	20.2		22.9	33.3	23.8		25.3	36.9	17.9		26.5	33.3	20.2		24.1	28.6	27.4	
Acute kidney injury	1.2	4.8	2.4		3.6	3.6	1.2		1.2	4.8	2.3		2.4	2.4	3.6		1.2	4.8	2.4	
Nephrolithiasis	3.6	2.4	1.2		6	0.0	1.2		3.6	2.4	1.2		3.6	2.4	1.2		3.6	2.4	1.2	
Multi causes	24.1	10.7	25		25.3	16.7	17.9		22.9	14.3	22.6		21.7	14.3	23.8		20.5	17.8	21.4	
Others	20.5	23.8	27.4		20.5	21.4	29.8		22.9	19	29.8		19.3	27.4	25		25.3	23.8	22.6	

Data are presented as mean ± SD for continuous and percent for categorical variables. *p*-value obtained from chi-square analysis for categorical variables and analysis of variance (ANOVA) for continuous variables. MUFA, monounsaturated fatty acid; PUFA, polyunsaturated fatty acid; SFA, saturated fatty acid; URR, urea reduction ratio.

**Table 2:** Nutrient intake across tertiles of different types of dietary fats.

	Total Fat				Cholesterol				Saturated fatty acid				MUFA				PUFA			
Nutrients	T1 (<40.18 gr/day)	T2 (40.18–58.84 gr/day)	T3 (≥58.84 gr/day)	*p* value	T1 (<132.69 mg/day)	T2 (132.69–213.22 mg/day)	T3 (≥213.22 mg/day)	*p* value	T1 (<12.59 gr/day)	T2 (12.59–20.11 gr/day)	T3 (≥20.11 gr/day)	*p* value	T1 (<13.97 gr/day)	T2 (13.97–20.61gr/day)	T3 (≥20.61gr/day)	*p* value	T1 (<7.49 gr/day)	T2 7.49–11.78 gr/day)	T3 ≥11.78 gr/day)	*p* value
Energy (Kcal/day)	1085.76 ± 347.95	1657.23 ± 376.71	2709.06 ± 1026.26	<0.001	1253.11 ± 498.22	1698.46 ± 579.29	2502.48 ± 1136.70	<0.001	1106.50 ± 378.39	1751.82 ± 461.33	2593.98 ± 1098.31	<0.001	1093.66 ± 369.68	1714.48 ± 428.86	2644.01 ± 1065.67	<0.001	1140.70 ± 384.63	1755.96 ± 578.98	2556.06 ± 1088.72	<0.001
Protein (g/d)	86.78 ± 11.78	61.68 ± 16.12	97.13 ± 31.48	<0.001	40.69 ± 15.30	60.80 ± 19.41	94.15 ± 34.12	<0.001	37.39 ± 12.51	66.01 ± 20.33	92.19 ± 34.08	<0.001	37.45 ± 12.77	62.13 ± 16.16	96.02 ± 32.82	<0.001	41.18 ± 15.31	65.63 ± 25.12	88.84 ± 35.14	0.003
Carbohydrate (g/d)	177.54 ± 72.71	251.77 ± 84.69	390.28 ± 137.65	0.791	203.11 ± 96.58	263.86 ± 109.17	352.93 ± 149.20	0.185	178.96 ± 77.30	269.37 ± 91.71	371.27 ± 147.95	0.681	177.61 ± 76.31	266.21 ± 92.58	375.77 ± 143.79	0.486	181.77 ± 73.91	267.46 ± 105.76	370.41 ± 142.46	0.605
Total fiber (g/d)	20.61 ± 9.22	31.16 ± 13.67	46.23 ± 17.00	0.17	23.64 ± 12.13	32.64 ± 14.49	41.75 ± 19.33	0.244	20.98 ± 9.26	33.23 ± 15.00	43.70 ± 18.00	0.067	20.25 ± 9.04	31.87 ± 12.77	45.88 ± 17.85	0.002	21.47 ± 9.46	32.98 ± 15.72	43.56 ± 17.69	0.074
Vitamin A (RAE)	272.64 ± 179.81	435.63 ± 238.06	785.68 ± 354.80	<0.001	304.61 ± 210.99	497.69 ± 283.44	692.04 ± 391.35	0.016	268.36 ± 168.54	460.16 ± 249.42	765.38 ± 369.01	<0.001	277.54 ± 183.19	454.50 ± 246.36	761.97 ± 371.61	<0.001	319.15 ± 217.7	485.17 ± 299.03	690.19 ± 383.62	0.574
Vitamin C (mg)	71.25 ± 42.77	132.09 ± 84.71	231.01 ± 140.78	0.838	95.05 ± 86.33	138.45 ± 8.82	201.12 ± 148.70	0.269	70.19 ± 42.40	143.17 ± 91.13	220.97 ± 142.74	0.468	70.19 ± 42.40	143.17 ± 91.13	220.97 ± 142.74	0.468	80.60 ± 50.72	148.18 ± 104.93	205.67 ± 143.04	0.189
Vitamin E (mg)	6.70 ± 2.50	10.07 ± 3.80	16.64 ± 22.39	<0.001	8.10 ± 3.81	10.19 ± 4.70	15.13 ± 22.60	0.001	7.43 ± 3.35	10.66 ± 4.14	15.32 ± 22.66	<0.001	6.42 ± 2.36	10.41 ± 3.18	16.58 ± 22.47	<0.001	5.95 ± 1.92	9.73 ± 2.50	17.72 ± 22.16	0.203
Vitamin D (μg)	0.35 ± 0.32	0.63 ± 0.56	1.20 ± 0.96	0.047	0.35 ± 0.43	0.66 ± 0.58	1.17 ± 0.91	0.002	0.35 ± 0.33	0.63 ± 0.53	1.20 ± 0.97	0.005	0.37 ± 0.34	0.67 ± 0.60	1.14 ± 0.96	0.248	0.43 ± 0.44	0.67 ± 0.63	1.07 ± 0.95	0.763
Calcium (mg)	609.89 ± 270.23	1060.92 ± 493.66	1686.26 ± 653.87	<0.001	699.84 ± 362.93	1130.70 ± 552.18	1527.61 ± 742.84	<0.001	607.15 ± 275.62	1116.39 ± 530.58	1633.51 ± 667.22	<0.001	622.51 ± 277.48	1080.09 ± 442.74	1654.62 ± 721.93	<0.001	732.03 ± 386.25	1143.19 ± 593.09	1483.31 ± 740.17	0.178
Thiamin (mg)	0.92 ± 0.39	1.36 ± 0.44	1.99 ± 0.70	0.042	1.05 ± 0.47	1.40 ± 0.60	1.82 ± 0.73	0.402	0.93 ± 0.44	1.46 ± 0.49	1.88 ± 0.72	0.057	0.94 ± 0.42	1.40 ± 0.49	1.94 ± 0.70	0.136	0.98 ± 0.42	1.44 ± 0.59	1.85 ± 0.72	0.293
Riboflavin (mg)	0.91 ± 0.33	1.51 ± 0.49	2.42 ± 0.78	<0.001	0.99 ± 0.43	1.56 ± 0.56	2.29 ± 0.88	<0.001	0.89 ± 0.33	1.58 ± 0.52	2.36 ± 0.81	<0.001	0.92 ± 0.34	1.55 ± 0.47	2.37 ± 0.86	<0.001	1.06 ± 0.45	1.62 ± 0.70	2.16 ± 0.91	0.069
Niacin (mg)	10.85 ± 4.12	17.35 ± 6.29	25.90 ± 9.60	<0.001	12.38 ± 5.50	16.98 ± 6.79	24.76 ± 10.44	0.001	11.19 ± 4.55	19.27 ± 7.74	23.65 ± 10.16	0.002	10.95 ± 4.34	17.19 ± 6.11	25.97 ± 9.66	<0.001	11.71 ± 4.97	18.28 ± 7.60	24.12 ± 10.21	0.047
Phosphorus (mg)	680.57 ± 244.80	1085.23 ± 268.70	1723.15 ± 521.36	<0.001	760.08 ± 303.13	1095.52 ± 362.62	1634.29 ± 587.88	<0.001	679.04 ± 250.62	1148.96 ± 312.00	1660.92 ± 561.16	<0.001	696.56 ± 261.48	1109.16 ± 287.23	1683.42 ± 561.23	<0.001	767.03 ± 287.06	1156.81 ± 432.62	1566.14 ± 608.07	0.042

Data are presented as mean ± SD. p-value obtained from analysis of covariance (ANCOVA) adjusted for total calorie intake. MUFA, monounsaturated fatty acid; PUFA, polyunsaturated fatty acid; SFA, saturated fatty acid.

Results of the calorie adjusted linear regression between QOL and different types of dietary fats among HD patients are presented in Table 3. There was a negative association between QOL and total fat (B = −0.115; 95% CI: −0.187, −0.043), SFA (B = −0.416; 95% CI: −0.688, −0.143), MUFA (B = −0.227; 95% CI: −0.389, −0.065) and PUFA (B = −0.228; 95% CI: −0.401, −0.056) when types of dietary fats were individually included to the regression analysis. We did not observe any relation between cholesterol intake and the score of QOL. When all dietary fats were simultaneously entered into the analysis, the association between QOL and MUFA (B = 0.394; 95% CI: −0.243, 1.031) and PUFA (B = −0.537; 95% CI: −1.159, 0.084) were attenuated. Inversely, regression coefficient for SFA remained significant (B = −0.553; 95% CI: −0.968, −0.138).

**Table 3:** Linear regression between quality of life and different types of dietary fats among hemodialysis patients.

Independent variables	Coefficient (B)^a^	Standard Error	95% CI	*p* value	*R* ^2^
Total fat	−0.115	0.037	−0.187, −0.043	0.002	8.9%
Cholesterol	−0.005	0.010	−0.025, 0.014	0.602	6.2%
SFA	−0.416	0.138	−0.688, −0.143	0.003	8.7%
MUFA	−0.227	0.082	−0.389, −0.065	0.006	8.3%
PUFA	−0.228	0.088	−0.401, −0.056	0.010	8.1%
All types of fats					
Cholesterol	0.003	0.011	−0.019, 0.025	0.783	10.3%
SFA	−0.553	0.211	−0.968, −0.138	0.009	
MUFA	0.394	0.324	−0.243, 1.031	0.224	
PUFA	−0.537	0.316	−1.159, 0.084	0.090	

*p*-value obtained from liner regression. MUFA, monounsaturated fatty acid; PUFA, polyunsaturated fatty acid; SFA, saturated fatty acid.

a Adjusted for total calorie intake.

The risk of low QOL per one-unit increase in intake of different dietary fats is presented in Table 4. There was no significant relationship between risk of low QOL and all types of dietary fat except for cholesterol in crude model (OR = 0.998, 95% CI: 0.996–1.000; *p* = 0.018). After adjusting for energy intake, we observed a significant association between the risk of low QOL and one-unit increase in intake of total fat (OR = 1.013, 95% CI: 1.004–1.022; *p* = 0.004), SFA (OR = 1.040, 95% CI: 1.006–1.076; *p* = 0.021), MUFA (OR = 1.028, 95% CI: 1.008–1.049; *p* = 0.005), and PUFA (OR = 1.029, 95% CI: 1.008–1.051; *p* = 0.007). Also, when all dietary fats were simultaneously entered into the analysis, we could not detect any significant relation between risk of low QOL and intake of dietary fats. In fully adjusted model (Model 2), there was a significant association between the risk of low QOL and on-unit increase in intake of total fat (OR = 1.011, 95% CI: 1.002–1.020), SFA (OR = 1.038, 95% CI: 1.004–1.074), MUFA (OR = 1.024, 95% CI: 1.004–1.045), and PUFA (OR = 1.024, 95% CI: 1.003–1.046). In Model 2 and after including all dietary fats simultaneously, the relation between risk of low QOL and MUFA and PUFA was attenuated and only a marginally significant association between SFA and the risk of low QOL was observed (OR = 1.051, 95% CI: 0.998–1.104).

**Table 4:** The Risk of low quality of life per one-unit increase of different dietary fats among hemodialysis patients.

	Crude		Model 1		Model 2	
Independent variables	Odds ratio (95% CI)	*p* value	Odds ratio (95% CI)	*p* value	Odds ratio (95% CI)	*p* value
Total fat (g/d)	0.994 (0.988, 1.001)	0.103	1.013 (1.004, 1.022)	0.004	1.011 (1.002, 1.020)	0.017
Cholesterol (mg/d)	0.998 (0.996, 1.000)	0.018	1.000 (0.998, 1.003)	0.729	1.001 (0.998, 1.003)	0.661
SFA (g/d)	0.981 (0.963, 1.001)	0.056	1.040 (1.006, 1.076)	0.021	1.038 (1.004, 1.074)	0.029
MUFA (g/d)	0.989 (0.973,1.006)	0.201	1.028 (1.008, 1.049)	0.005	1.024 (1.004, 1.045)	0.020
PUFA (g/d)	0.993 (0.977, 1.010)	0.429	1.029 (1.008, 1.051)	0.007	1.024 (1.003, 1.046)	0.025
All types of fats						
Cholesterol (mg/d)	0.998 (0.996, 1.001)	0.235	1.00 (0.997, 1.002)	0.751	1.000 (0.997, 1.002)	0.848
SFA (g/d)	1.007 (0.962, 1.054)	0.763	1.051 (1.00, 1.104)	0.051	1.050 (0.998, 1.104)	0.058
MUFA (g/d)	0.965 (0.896, 1.039)	0.346	0.971 (0.899, 1.049)	0.455	0.968 (0.896, 1.046)	0.408
PUFA (g/d)	1.038 (0.965, 1.117)	0.311	1.052 (0.976, 1.134)	0.186	1.051 (0.975, 1.134)	0.196

*p*-value obtained from logistic regression. Model 1: Adjusted for energy intake. Model 2: Adjusted for energy intake, sex, age and dialysis quality. MUFA, monounsaturated fatty acid; PUFA, polyunsaturated fatty acid; SFA, saturated fatty acid.

## Discussion

We found that dietary intake of SFA, MUFA, PUFA and total fat was inversely associated with QOL. Nevertheless, the relation between QOL and MUFA and PUFA was attenuated when SFA was simultaneously entered into the regression analysis. These findings revealed that the relation between QOL and MUFA, PUFA and total fat was mediated by SFA.

Over the past decades, sources of dietary fat in the general population have changed significantly. The main change is substituting PUFA and MUFA for SFA (21). There is a consensus that PUFA and MUFA is healthy lipids due to their ability to decrease the risk of cardiovascular diseases (25). However, SFA is recognized as a major CVD risk factor (26). Moreover, higher intake of SFA has been linked to declined cognitive function (46) and increased risk of depression (47), anxiety (32) and dementia (48).These pieces of evidence support our findings that SFA intake was negatively associated with QOL. It is suggested that the impact of changes in dietary fat on QOL in different diseases should be assessed in future studies.

We found that the negative association between QOL and MUFA or PUFA was mediated by SFA and results were insignificant when SFA was simultaneously entered into the regression analysis. Foods containing fats typically contain a wide range of fatty acids, including SFA, MUFA and PUFA (49). SFAs are found in both plant and animal food sources. Animal-source foods such as red meat, bacon, lard, and high-fat dairy products are condensed sources of SFA (50). On the other hand, oils extracted from palm kernel, coconut, olive, soybean and sunflower are considered plant sources of SFA (51). For instance, the concentration of SFA in olive oil may be up to 25% (52). Therefore, there is a co-consumption of MUFA/PUFA and SFA; subjects who consumed more vegetable oil may intake large amounts of SFA.

Consequently, assessment of dietary intake by questionnaires cannot determine the exact intake of different types of dietary fats, and more reliable methods such as dietary biomarkers (e.g., plasma phosphor acid fatty acid composition and adipose tissue) should be used (53), Evidence showed that using biomarkers may alter the conclusion of a study. A meta-analysis reported that blood concentration of PUFA was inversely associated with the risk of cancer. However, there was no significant association between dietary intake of PUFA and the risk of cancer (54).

We found an indirect association between SFA intake and QOL. Metabolic changes that occurred after consuming various types of fatty acids may explain our finding. High dietary SFA intake elevates inflammation by stimulating the secretion of proinflammatory compounds (e.g., TNF -α and IL-6) and reducing anti-inflammatory cytokines such as interleukin-10 and Arginase-1 (55).

Previous findings indicated that chronic inflammation could negatively affect the emotional and social domains of QOL (56). Therefore, the association between high dietary intake of SFA and decreased QOL is reasonable. In addition, a high intake of dietary SFAs promotes insulin resistance by releasing inflammatory cytokines and endotoxins (57). Insulin resistance and impaired glucose regulation can result in neuronal dysfunctions and lead to cognitive deficits (58–60).

The limitations of this study should also be addressed. First, we could not evaluate the relationship between *trans* fatty acid and QOL because we had no data regarding the *trans* fatty acid content of the foods. Second, we did not assess dietary intake by a biomarker. Third, the cross-sectional design of our study had several limitations regarding a causal relationship between dietary SFA intake and QOL.

## Conclusion

In conclusion, among different types of dietary fats, we found an inverse relationship between dietary intake of SFA and QOL. Also, the relation between QOL and MUFA, PUFA and total fat was mediated by SFA.

In future studies, a specific focus should be placed on the type of fat intake as a key factor affecting the patient’s quality of life. Furthermore, to evaluate the quantity and type of consumed fat in such studies, a suitable biomarker is recommended. It is hoped that the association between types of fat intake and symptoms and complications of this disease will be evaluated comprehensively to improve the guidelines and recommendations for HD patients.

## Data Availability

The raw data supporting the conclusions of this article will be made available by the authors, without undue reservation.
